# Chemical Composition, Antimicrobial activity, In Vitro Cytotoxicity and Leukotoxin Neutralization of Essential Oil from *Origanum vulgare* against *Aggregatibacter actinomycetemcomitans*

**DOI:** 10.3390/pathogens9030192

**Published:** 2020-03-05

**Authors:** Sanae Akkaoui, Anders Johansson, Maâmar Yagoubi, Dorte Haubek, Adnane El hamidi, Sana Rida, Rolf Claesson, OumKeltoum Ennibi

**Affiliations:** 1 Research laboratory in oral biology and biotechnology, Faculty of dental medicine, Mohammed V University in Rabat, Rabat 10 000, Morocco; sanae.akkaoui@um5s.net.ma; 2 Division of Molecular Periodontology, Department of Odontology, Umeå University, 901 87 Umeå, Sweden; anders.p.johansson@umu.se; 3 Microbiology Laboratory, faculty of medicine and pharmacy, Mohammed V University in Rabat, Rabat 10 000, Morocco; m.yagoubi@um5s.net.ma; 4 Section for Pediatric Dentistry, Department of Dentistry and Oral Health, AarhusUniversity, 8000 Aarhus, Denmark; dorte.haubek@dent.au.dk; 5 Materials, Nanotechnologies and Environment laboratory, Faculty of Sciences, Mohammed V University in Rabat, Rabat 10 000, Morocco; adnane_el@hotmail.com; 6 Department of endodontics, Research laboratory in oral biology and biotechnology, Faculty of Dental Medicine, Mohammed V University in Rabat, Rabat 10 000, Morocco; s.rida@um5s.net.ma; 7 Division of Oral Microbiology, Department of Odontology, Umeå University, 901 87 Umeå, Sweden; rolf.claesson@umu.se; 8 Department of Periodontology, Research laboratory in oral biology and biotechnology, Faculty of Dental Medicine, Mohammed V University in Rabat, Rabat 10 000, Morocco

**Keywords:** *Aggregatibacter actinomycetemcomitans*, periodontitis, *Origanum vulgare*, essential oil, antimicrobial activity, minimum inhibitory concentration, minimal bactericidal concentration, cytotoxicity, leukotoxin neutralization

## Abstract

In this study, the essential oil of *Origanum vulgare* was evaluated for putative antibacterial activity against six clinical strains and five reference strains of *Aggregatibacter actinomycetemcomitans*, in comparison with some antimicrobials. The chemical composition of the essential oil was analyzed, using chromatography (CG) and gas chromatography–mass spectrometry coupled (CG–MS). The major compounds in the oil were Carvacrol (32.36%), α-terpineol (16.70%), *p*-cymene (16.24%), and Thymol (12.05%). The antimicrobial activity was determined by an agar well diffusion test. A broth microdilution method was used to study the minimal inhibitory concentration (MIC). The minimal bactericidal concentration (MBC) was also determined. The cytotoxicity of the essential oil (IC50) was <125 µg/mL for THP-1 cells, which was high in comparison with different MIC values for the *A. actinomycetemcomitans* strains. *O. vulgare* essential oil did not interfere with the neutralizing capacity of *Psidium guajava* against the *A. actinomycetemcomitans* leukotoxin. In addition, it was shown that the *O. vulgare* EO had an antibacterial effect against *A. actinomycetemcomitans* on a similar level as some tested antimicrobials. In view of these findings, we suggest that *O.vulgare* EO may be used as an adjuvant for prevention and treatment of periodontal diseases associated to *A. actinomycetemcomitans*. In addition, it can be used together with the previously tested leukotoxin neutralizing *Psidium guajava*.

## 1. Introduction

*Aggregatibacter actinomycetemcomitans* is a capnophilic Gram-negative coccobacillus, widely known as one of the putative pathogens associated with periodontitis, mainly in adolescents and young adults [1]. Pathogenesis of periodontitis is very complex, including immunogenetic factors, life style, proportion, and composition of specific periodontitis associated bacterial species, including *A. actinomycetemcomitans* in the oral biofilm [2]. The contribution of *A. actinomycetemcomitans* in initiation and progression of the disease is due to various virulent factors released in periodontal tissues [3]. A total of seven serotypes (a,b,c,d,e,f, and g) [2,4] of *A. actinomycetemcomitans* have been isolated from periodontal lesions worldwide. Patients seem to be colonized by a single serotype for life [2]. Among virulence factors of the bacterium, the leukotoxin (LtxA) is the most studied [3,5]. It activates or kills immune cells, helping the bacterium to survive in a site of infection. A specific variant of *A. actinomycetemcomitans* produces more LtxA than other variants [6]. This highly leukotoxic clone, JP2, has been associated with aggressive forms of periodontitis in Morocco [7,8]. According to the 2017 World Workshop of Periodontology, aggressive periodontitis is included in the category of “periodontitis”, which is characterized based on stages and grades. Extension and distribution of periodontal lesions allow distinguishing localized, generalized, and molar-incisor distribution forms [9]. 

Aggressive periodontitis treatment of is based on mechanical debridement with antimicrobials as adjuvants. This aims to allow the elimination of *A. actinomycetemcomitans* and other bacterial species which penetrate the periodontal epithelial tissue [10]. However, given the increasing resistance of oral bacteria to antimicrobials and the side effects caused by antiseptic agents often used in dentistry (i.e., dental staining and taste alteration) [11], the search for new natural agents as alternative therapeutic products with fewer side effects (e.g., gastric problems) and less bacterial-resistance development has become a necessity. 

Morocco has, by its geographical diversity, great natural resources for cultivation of medicinal and aromatic plants. Surveys performed in Morocco among the population in different regions have shown a frequent therapeutic use of this natural heritage in traditional medicine [12,13].

Many studies through the world have been carried out to screen medicinal and pharmacological properties of different plants and essential oils, to integrate them into the therapeutic arsenal, according to standards of quality and effectiveness [14,15].

However, the use of medicinal plants and essential oils is limited in dentistry, and their antimicrobial activities on oral bacteria are not widely studied. We have previously reported that, in Moroccan population, *O.vulgare* is used as a mouthwash in traditional medicine [13].

*O. vulgare* is a widespread aromatic plant naturally growing in different parts of the world, including Northern Africa, the Mediterranean area, the Arabian Peninsula, Central Asia, and Europe [16,17]. It belongs to Lamiaceae family, and it is known for being a rich source of EOs, which have proven to possess a large variety of biological activities because of their chemical compounds [18]. The abundance of different compounds of EOs of Origanum species may show some variations because of ecological and environmental effects, geographical location, and time of collection [19,20,21].

We have additionally evaluated the possibility to neutralize the LtxA by administrating a mouth rinse with LtxA neutralizing agents released from leaves of *Psidium guajava* [22].

The aim of the present work was to study the antibacterial activity of *O. vulgare* EO of Moroccan origin on *A. actinomycetemcomitans*. In addition, we explored its cytotoxicity and checked how it cooperates with the leukotoxin neutralizing properties.

## 2. Results

### 2.1. Chemical Composition of the Essential Oil

**Figure 1:**
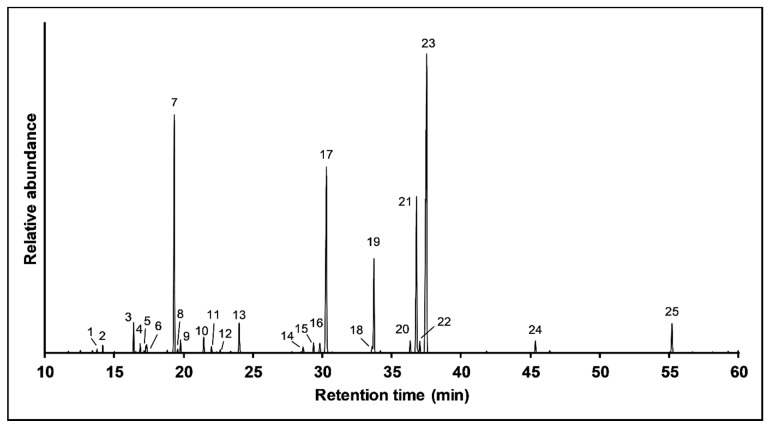
Chromatography/mass spectrometry (GC/MS) chromatogram of *O.vulgare*EO.

The chemical analysis of *O. vulgare* EO was performed by using gas chromatography/mass spectrometry (GC/MS) technique. The 25 identified components and their relative percentage are summarized in Table 1. The major constituents were as follows: Carvacrol (32.36%), α-terpineol (16.70), *p*-cymene (16.25%), and Thymol (12.06%) (Figure 1). Thus, the EO of *O. vulgare* is dominated by oxygenated monoterpenes. 

### 2.2. Antimicrobial Activity

The inhibition zones obtained respectively when the *O. vulgare* EO, Amoxicillin (AM), Amoxicillin and clavulanic acid (AMC), and Doxycycline (DO) were tested against six clinical strains and five reference strains of *A. actinomycetemcomitans* are shown in Table 2. All tested strains showed inhibition zones in the presence of the EO (27.6 µg) (37–69 mm). The susceptibility to the antimicrobials varied among the strains (Table 2).

The serial diffusion test in 96-well microplates showed MIC values in the ranges of 0.05 to 1.51 μg/mL and 0.09 to 2.01 μg/mL for MBCs (Table 3). For MBC/MIC ratio, all the values found were lower than 4, considering EO as bactericidal agents.

### 2.3. Cytotoxicity

**Figure 2:**
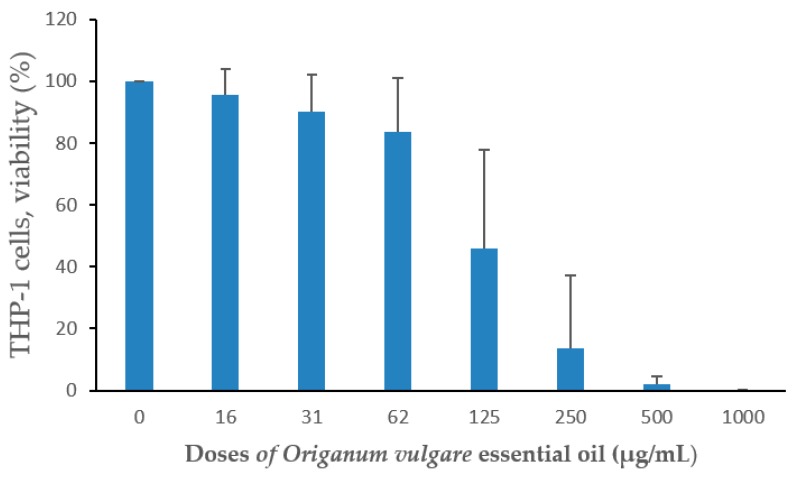
Cytotoxicity of *O.vulgare* essential oil.

The *O. vulgare*EO showed a dose-dependent cytotoxic effect in cultures of PMA-differentiated THP-1 cells (Figure 2). Concentrations of oil above 125 µg/mL causedsubstantial decreased cell viability after 24 h of exposure.

PMA-differentiated THP-1 cells were exposed to different concentrations of oil for 24 h and cell viability determined with neutral red uptake staining. Mean ± standard deviation of three independent experiments are shown.

### 2.4. A. actinomycetemcomitans Leukotoxin Neutralization

The *O. vulgare* EO showed no neutralizing effect on *A. actinomycetemcomitans* leukotoxicity estimated in cultures of PMA-differentiated THP-1 cells (Table 4). The presence of oil from *O. vulgare* did not affect the inhibitory effect on leukotoxicity exhibited by the extract from the *Psidium guajava* leaves.

## 3. Discussion

The use of natural products, such as Eos, as antibacterial agents is expanding in oral hygiene and dentistry. In Morocco, a major herbal-producing nation, many studies have been carried out on the antimicrobial activity of Moroccan plant extracts and EOs [23,24].

However, there are few reports on the effects of Moroccan EOs on periodontal pathogens. The EO of *O. vulgare* was selected for this study on the basis of its traditional use for the treatment of oral diseases [13].

The results of the present study showed that the essential oil of *O. vulgare* exhibited a strong antimicrobial activity against all tested clinical and reference strains of *A. actinomycetemcomitans*.

The antimicrobial activity of an EO is well-known, and it is linked to its main constituents. The *O. vulgare* EOs is characterized by the dominance of various antibacterial compounds. Chemical analyses of this oil revealed that the main constituents of the oil were carvacrol 32.36%, α-terpineol 16.70%, p-cymene 16.24%, and thymol 12.05% (Figure 1 and Table 1). All these compounds have strong antibacterial activity, as shown in previous studies [25,26,27]. Linalool (an alcohol) is one of the components of the essential oil of *O. vulgare* present in lower concentration (Table 1). However, this compound was found to have antimicrobial activity against various oral and non-oral microbes [28,29,30].

In this study, we used the agar diffusion test to study the antibacterial activity of *O. vulgare* EO against clinical and reference strains of *A. actinomycetemcomitans*. Substantial antibacterial activity against both JP2 and non-JP2 strains was observed. The diameters of the inhibition zones obtained were greater than 20 mm, (going from 37.00 ± 1.73 mm for strain HK 921(JP2), to 69.66 ± 0.57 mm for strain ATCC 43717 Sunny aB75) (Table 3). The studied strains showed a high sensitivity to the studied essential oil when 27.6 µg of it was used.

For analyzing the susceptibility of the *A. actinomycetemcomitans* strains to the tested antimicrobials, and according to the EUCAST (European Committee on Antimicrobial Susceptibility Testing), breakpoints for *Haemophilus influenzae* was used, as there is not one associated to *A. actinomycetemcomitans* [31].

Based on the EUCAST interpretation values, all 11 tested *A. actinomycetemcomitans* strains were susceptible to AMC and MH. All isolates were also susceptible to AML if ampicillin-derived breakpoints were used. Furthermore, eight strains were susceptible to DO if tetracycline-derived breakpoints were used. Susceptibility to SP, VA and MTZ could not be validated, since no breakpoints for *H. influenzae* are available in the EUCAST database. Inhibition zones are usually absent when susceptibility of *A. actinomycetemcomitans* to MTZ is tested under aerobic conditions. 

For studying the antibacterial effect of the essential oil to the selected *A. actinomycetemcomitans* strains, compared with corresponding effect of the antimicrobials, the inhibition zones were used. Since all inhibition zones induced by the essential oil (27.6 µg) were significantly larger, it indicates that the oil has a substantial antibacterial effect on *A. actinomycetemcomitans*. 

A range of different antimicrobials have been used as adjuvant for treatment of periodontitis during the last decades. This has raised questions about risk for resistance development among the antimicrobials and also doubts about the beneficial value of this treatment strategy. However, a range of clinical studies have shown that use of a combination of MTZ and AML in conjunction with standard periodontal treatment of aggressive forms of periodontitis achieves better clinical and microbiological results than treatment without these antimicrobials [32,33,34].

Regarding resistance development among these antimicrobials, different results are reported. However, the breakpoint for MTZ is not available. Thus, susceptibility testing of this antimicrobial is not relevant. Based on usage of breakpoint value for *Haemophilus influenzae,* most reports of susceptibility of *A. actinomycetemcomitans* to AML show that the prevalence of resistant isolates of the bacterium is low [35]. In addition, when isolates earlier found to be resistant to this antimicrobial were retested, the opposite result was achieved [36].

For the microdilution test, the results obtained for MIC are mainly in accordance with the diameters of the inhibition zones observed in the well diffusion test. On the other hand, the *O. vulgare* EO showed bactericidal activity with promising MBC results and an MBC/MIC ratio below 4. The MBC values were similar or almost identical to those of MIC. *O. vulgare* EO had the highest inhibitory activity against ATTC 43717 (Suny aB75) (CMI = 0.09 μg/mL) and the highest bactericidal effect against the reference strains ATTC 43717 (Suny aB75) and HK1651 (JP2) (CMB= 0.09 μg/mL) (Table 3). These results are consistent with those obtained in previous work on other tested non-oral Gram-negative bacteria [37,38,39] reflecting a higher antibacterial activity on this oral bacterium.

The cytotoxicity of the essential oil of *O. vulgare* EO *in* concentrations was below 125 µg/mL when analyzed in cultures of human macrophages (THP-1 cells). The IC50-value for the oil was much lower than the MIC-values for antibacterial effect, suggesting an advantage for its use as a clinical chemical agent. EO seems to be toxic for cells. Previously, it has been shown that carvacrol and other oregano constituents can be cytotoxic at high doses [40,41]. Thus, further investigations are needed to achieve maximum positive antimicrobial effect of *O.vulgare* EO without cytotoxic effects. In addition, this oil did not interfere with the LtxA neutralizing capacity of extract from *Psidium guajava* leaves. It has been shown that components extracted from *Psidium guajava* bind to LtxA and completely abolish its activity [42].

A mouth rinse containing water extract from *Psidium guava* leaves has been tested on adolescents in Morocco with the presence of *A. actinomycetemcomitans* from the JP2 genotype in their subgingival plaque [22]. Results from this pilot study are limited, but they indicate that LtxA neutralization alone was not sufficient for eradication of *A. actinomycetemcomitans* and its pro-inflammatory effect.

*A. actinomycetemcomitans* is a germ that is mostly associated with aggressive forms of periodontitis. One of its virulence factors is LtxA, which plays an important role in pathogenicity. Periodontal infections due to strains that produce high levels of LtxA are strongly associated with a serious disease. LtxA selectively kills human leukocytes and can affect the body’s functioning local defensive mechanisms [5]. Previous studies on the role of LtxA in host–parasite interactions have focused mainly on polymorphonuclear leukocytes (PMN) [43,44]. In periodontal inflammation, macrophages have an important role in regulating inflammatory reactions and tissue degradation and remodeling [45]. LtxA causes a rapid inflammatory cell death in macrophages, which might cause an imbalance in the pro-inflammatory response [46].

We can conclude that the *O. vulgare* EO has the potential to be used as a preventive or therapeutic agent against periodontitis in individuals colonized with *A. actinomycetemcomitans*. Its cytotoxic properties can be overcome by the possibility to cooperate with the LtxA neutralizing compounds of *Psidium guajava*.

## 4. Materials and Methods

The present study was carried out after obtaining approval from the Biomedical Ethics Committee (Ref. 400/2010); the individual patient’s written informed consent was obtained before the collection of the plaque samples for the study.

### 4.1. Plant Material and Extraction of Essential Oil

The aerial part of *O. vulgare* was purchased at a local market in Rabat, Morocco. A portion (100 g) of the aerial parts of the plants was hydrodistilled during 3 hours, using a Clevenger system. The obtained essential oil was dried over anhydrous sodium sulphate and, after filtration, stored at +4 °C, until it was tested and analyzed.

### 4.2. Gas Chromatography Coupled with Mass Spectrometry (GC/MS)

The chemical composition of the EO was analyzed by using a gas chromatograph (Perkin Elmer Clarus^TM^ GC-680) fitted to a mass spectrometer (Q-8 MS Ion Trap), operating in electron-impact EI (70 eV) mode. Non-polar column HP-5MS (Methylpolysiloxane 5% phenyl, 60 m × 0.25 mm × 0.25 μm thickness) was used (the GC/MS was done atthe Platform of physicalchemistry analysis and characterization, Faculty of Sciences, Mohammed V University in Rabat, Morocco). The chromatographic conditions were as follows: injector temperatures at 280 °C; carrier gas, helium at flow rate of 1 mL/min; temperature program ramp from 60 to 200 °C, at gradient of 2 °C/min. After holding 1 min at 200 °C, another ramp was operated from 200 until 300 °C, at 20 °C/min, and final hold for 5 min. The GC/MS system was controlled by Turbomass^TM^software; a library search was carried out, using the combination of NIST MS Search and literature. The NIST version was 2.0 g, built May 19, 2011. The relative number of individual components of the total oil was expressed as a percentage of each peak area relative to total peak areas. The retention indices (RI) were obtained by injecting in HP-5MS a mixture of continuous series of straight chain hydrocarbons (C8-C31), under the same conditions as described above.

### 4.3. A. actinomycetemcomitans Strains 

The antimicrobial capacity of *O.vulgare* EO and of following selected antimicrobials, Amoxicillin (AM), Amoxicillin and clavulanic acid (AMC), Doxycycline (DO), Ciprofloxacin (CIP), Minocycline (MN), Vancomycin (VA), and Metronidazole(MTZ) was tested against six clinical isolates and the following reference strains: ATTC 43717 (Suny aB75), Y4 (ATCC 43718),HK1651(JP2),HK 921 (JP2), and HK1605 (non JP2). HK 1651 was obtained from Department of Odontology, Umeå University, Sweden. HK 921 (JP2) and HK1605 (non JP2) were obtained from Department of Dentistry and Oral Health Aarhus University, Denmark.

The bacterial strains and essential oil were stored at the laboratory of oral biology and biotechnology, Faculty of Dental Medicine, Mohammed V University in Rabat. The tests were performed at the same laboratory.

### 4.4. Subgingival Plaque Sampling 

The clinical *A. actinomycetemcomitans* strains were obtained from subgingival plaque samples collected from patients with periodontitis. The patients were recruited at the Clinical Department of Periodontology in the Center of Consultations and Dental Treatments (CCTD) in Rabat-Morocco.

The sampled patients were diagnosed with aggressive periodontitis and had pockets of 5 mm or greater confirmed by clinical and radiological examination. Subgingival sampling of periodontal biofilm was performed, using absorbent paper point (medium size, Maillefer, Ballaigues, Switzerland). Then, the papers were pooled and placed in a tube containing 1.5 mL of phosphate buffer saline (PBS). All plaque samples were collected by the same examiner.

### 4.5. Culture and Isolation

Once in the laboratory, the sample was vortexed before being seeded into the culture medium Dentaid-1 [47] and used for selective isolation and growth of *A actinomycetemcomitans*. Plates were incubated at 37 °C, in air, with 5% CO_2_; and after 3–5 days, they were carefully examined for the presence of *A. actinomycetemcomitans*. Identification of the bacterium was based on colony morphology, positive catalase reaction, and negative oxidase reaction. Putative *A. actinomycetemcomitans* colonies were further elucidated by microscopy, Gram stain, and enzymatic activity, including indole and fermentation of glucose, xylose, maltose, and mannitol [48]. The isolates were then stored at −80 °C in glycerol broth.

### 4.6. In Vitro Antimicrobial Susceptibility Assay

The antimicrobial effect of *O. vulgare* was tested in two ways. The agar well diffusion method was used to determine the antibacterial activity in comparison with corresponding activity of selected antimicrobials. A microdilution assay was used for the calculation of MIC (minimum inhibitory concentration). MBC (minimal bactericidal concentration) of the oil was also determined. 

#### 4.6.1. Agar Well Diffusion Method

Agar well diffusion method [49,50,51] was used to evaluate the antimicrobial activity of essential oil from *O. vulgare.*

Initially, the bacterial strains were cultivated on slant cultures for 24 hours. Subsequently, bacterial suspensions were prepared in 0.85% NaCl, and the turbidity was adjusted to McFarland 0.5 (approximately 1 × 10^8^ CFU/mL). The turbidity was confirmed by a Sensititre® Nephelometer. At first, the agar plates were seeded by swabbingwith a cotton swab. Then, 30 μLof the essential oil (27.6 µg) was added to wells (diameter 6 mm) made in the center of each agar plate after 15 minutes [52,53]. Doxycycline (disc: 30 μg) was used as positive control [54]. The plates were incubatedat 37 °C, in an aerobic atmosphere containing 5% CO_2_, for 48 hours. All tests were carried out in triplicate, in separate experiments. Diameters of the inhibition zones were measured as (mm), including the diameter of the well. The antibacterial activity was considered if an inhibition halo of growth larger than 6 mm (size of the well) was produced.

#### 4.6.2. Minimum Inhibitory Concentration (MIC) Determination

The MIC of *O. vulgare* EO was determined by using a broth assay in 96-well microplates (Sigma-Aldrich, USA), as recommended by NCCLS, for the determination of the MIC (NCCLS, 1999). All tests were performed in Mueller Hinton broth (MHB) supplemented with Tween 80, (final concentration of 0.5% v/v), aimed to improve the solubility. The bacterial strains were cultured overnight, at 37°C, in Dentaid-1. The turbidity of the inoculums was adjusted to McFarland 0.5. The turbidity of the suspensions was confirmed with the Sensititre® Nephelometer. Serial dilutions ranging from 48.42 to 0.09 mg/mL of the EO were prepared in a 96-well microplate, including one growth control with “MHB + Tween 80”, one sterile control containing “MHB and Tween 80”, and another sterile control made of “MHB +Tween 80 +EO”. Amoxicillin (10 mg/mL) was used as positive control. The plates were incubated under normal atmospheric conditions, at 37 °C, for 24 hours. After incubation time, 40 µL of 2 mg/mL Triphenyl Tetrazolium Chloride (TTC) indicator solution (indicator of microorganism growth) was added to all wells of the microplate. Subsequently, the plates were re-incubated for 2 hours, at 37 °C [55]. Bacterial growth was monitored when the TTC indicator was red.

#### 4.6.3. Minimum Bactericidal Concentration (MCB) Determination

To measure the minimum bactericidal concentration (MBC), 10 µL of cultures was taken from wells of the microplate of MIC, with no visible turbidity, inoculated on blood agar plates, and incubated for 48 hours, at 37 °C, under 5% CO_2_ [56]. MBC was defined to be the lowest concentration of essential oil that killed 99.9% of the microorganisms in culture on the agar plate after the incubation time. 

The MBC/MIC ratio was calculated to show the nature of the antibacterial effect of essential oils. In a ratio less than 4, the essential oil was classified as a bactericidal essential oil, and in a ratio more than 4, it was classified as a bacteriostatic essential oil [57]. 

Each MIC and MBC value was obtained from three independent experiments.

### 4.7. Cell Culture

Cells of the human acute monocytic leukemia cell line THP-1 (ATCC 16) were cultured in RPMI-1640 (Sigma-Aldrich, St. Louis, MI, USA) with 10% fetal bovine serum (FBS) (Sigma-Aldrich), at 37 °C, in 5% CO_2_. Before determination of leukotoxic activity, the THP-1 cells were seeded in 96-well cell-culture plates, at a cell density of 10^5^ cells/mL in 100 μL culture medium supplemented with 50 nMphorbol 12-myristate 13-acetate (PMA, Sigma-Aldrich), and incubated for 24 hours. The PMA-activated THP-1 cells exhibited adherent properties and enhanced sensitivity to the LtxA.

After differentiation with phorbol 12-myristate 13-acetate (PMA), THP-1 cells acquire a macrophage phenotype, which is similar to primary human macrophages in many aspects [58,59]. This is the common way to use THP-1 cells in cell assays. The adherent phenotype is macrophage-like and easier to study in cytotoxicity assays.

Twenty-four hours prior to LtxA exposure, the culture medium was discarded, and 100 μL fresh medium without PMA was added to each well of the THP-1 monolayer.

### 4.8. Cytotoxicity Assay

The cell monolayers of PMA-differentiated THP-1 cells were exposed to different concentrations of the oil for 24 h, at conditions described above. The proportion of viable cells in each well was determined by the neutral red uptake method and expressed in relation to that of the control cells cultured in plane medium [60]. Cytotoxicity (LD_50_) was expressed as the lowest concentration (ppm) that kills ≥ 50% of the cells.

### 4.9. LtxA Purification

LtxA was purified from *A. actinomycetemcomitans* strain HK 1519, described in detail previously [61]. The purified LtxA was basically free from lipopolysaccharides (LPS) (<0.001% of total protein) and visualized by SDS-polyacrylamide gel separation.

### 4.10. Preparation of Psidium Guajava Leave Extract

Guava leaves were collected in Ghana by Dr. F.Kwamin and transported with a courier to Umeå University, Umeå, Sweden. Guava leaves at a concentration of 250 g/liter of water, were boiled for 10 minutes before the leaves were removed by filtration, and cleared from debris by centrifugation. The supernatant was aliquoted and stored in a refrigerator until use. The amount of dry substance was determined by evaporation of the extract, which contained 10.0 mg/mL H_2_O.

### 4.11. *LxtA* Neutralization Assay

The cell monolayers of PMA-differentiated THP-1 cells were exposed to different concentrations of the oil or guava for 15 min, before the LtxA (200 ng/mL) was added. The different mixtures were incubated for 2 h, at conditions described above. The proportion of viable cells in each well was determined by the neutral red uptake method, as described above.

### 4.12. Statistical Analyses

Statistical analysis was carried out by using SPSS for Windows (SPSS, Inc., Chicago, IL, USA). Inhibition zone diameter, MIC, MBC, MBC/MIC, and cell viability values, as continuous variables with a normal distribution, were expressedas mean ± standard deviation. For statistical differences between the inhibition diameter of the nine antimicrobial agents (EO, AMC, AMX, DO, SP, CIP, MH, VA, and MTZ), andthe cell viability in presence of EO, Guava, and the association “EO +Guava”, the One-Way Analysis of Variance (ANOVA) with Bonferroni correction was performed. A *p*-value < 0.05 was considered as statistically significant. Statistical analyses were carried out, using SPSS for Windows (SPSS, Inc., Chicago, IL, USA).

## 5. Conclusions

The present study indicates that *O. vulgare* EO may find application as an antibacterial agent on periodontitis associated with *A. actinomycetemcomitans* and shows the possibility of *Psidium guajava* to overcome its cytotoxic properties. However, further investigations on mechanisms of action and toxicity need to be continued. 

## Figures and Tables

**Table 1:** Chemical composition of *O. vulgare*EO.

No	Compound	Formula	RT (min)	RI	Conc. (%)
1	α-Thujene	C_10_H_16_	13.768	927	0.20
2	α-pinene	C_10_H_16_	14.179	936	0.44
3	1-octen-3-ol	C_8_H_16_O	16.403	942	1.69
4	3-Octanone	C_8_H_16_O	16.877	989	0.53
5	Dehydrocineole	C_10_H_16_O	17.291	995	0.39
6	3-Octanol	C_8_H_18_O	17.351	997	0.42
7	*p*-cymene	C_10_H_14_	19.335	1028	16.25
8	Limonene	C_10_H_16_	19.575	1031	0.28
9	Eucalyptol	C_10_H_18_O	19.782	1035	0.82
10	γ-terpinene	C_10_H_16_	21.457	1061	1.03
11	Cis-Sabinene hydrate	C_10_H_18_O	22.01	1068	0.43
12	1-hepten-3-ol	C_7_H_14_O	22.641	1084	0.21
13	Linalool	C_10_H_18_O	24.005	1101	2.16
14	Borneol	C_10_H_18_O	28.615	1167	0.37
15	Terpinen-4-ol	C_10_H_18_O	29.372	1179	0.77
16	*p*-Cimen-8-ol	C_10_H_14_O	29.822	1185	0.82
17	α-terpineol	C_10_H_18_O	30.296	1189	16.70
18	Pulegone	C_10_H_16_O	33.591	1251	0.49
19	Thymol methyl ether	C_11_H_16_O	33.721	1235	6.60
20	*p*-Cymen-3-ol	C_10_H_14_O	36.33	1287	0.88
21	Thymol	C_10_H_14_O	36.787	1290	12.06
22	*p*-Thymol	C_10_H_14_O	37.024	1291	0.74
23	Carvacrol	C_10_H_14_O	37.524	1300	32.36
24	Caryophyllene	C_15_H_24_	45.356	1418	0.93
25	Caryophyllene oxide	C_15_H_24_O	55.195	1582	2.42

RT: retention time; RI: retention index; Conc.: Concentration

**Table 2:** Mean diameter of inhibition zones (mm) obtained by the agar diffusion method and the interpretation according to the European Committee on Antimicrobial Susceptibility Testing Breakpoint tables for interpretation of MICs and zone diameters Version 9.0, valid from 2019-01-01.

A. a. strains	Inhibition Zone Diameter (mm) ^1*^									*p*-value
	EO	Antimicrobials								
	*O. vulgare* 27.6 µg	AMC ^2^	AML ^3^	DO ^4^	SP ^5^	CIP ^6^	MH ^7^	VA ^8^	MTZ ^9^	
		25 µg	25 µg	30 µg	100 µg	5 µg	30 µg	30 µg	5 µg	
		Breakpoints								
		S ≥ 15 R < 15	note^10^	note^11^	no info	S ≥ 30 R < 30	S ≥ 24 R < 21	no info	no info	
clinical strain 1	37.33 ± *2.08*	31.66 ± *1.15*	30.33 ± *0.57*	25.00 ± *0.00* **	29.66 ± *0.57*	28.33 ± *0.57*	36.00 ± *1.73*	*0.00*	*0.00*	*<0.001*
	***S***	***S***	***S***	***S***	--	***R***	***S***	--	*R*	
clinical strain 2	51.33 ± *0.57* **	32.66 ± *0.57* **	29.33 ± *1.15* **	23.33 ± *0.57*	22.66 ± *0.57*	26.66 ± *0.57* **	36.33 ± *0.57* **	*14.33 ± 0.57* **	*0.00*	*<0.001*
	***S***	***S***	***S***	***S***	--	***R***	***S***	--	*R*	
clinical strain 3	65.66 ± *0.57* **	30.66 ± *0.57*	30.66 ± *1.15*	25.66 ± *0.57*	28.66 ± *1.15*	29.33 ± *1.15*	38.33 ± *2.08* **	*15.66 ± 0.57* **	*0.00*	*<0.001*
	***S***	***S***	***S***	***S***	--	***R***	***S***	--	*R*	
clinical strain 4	63.66 ± *0.57* **	32.00 ± *1.00*	28.66 ± *1.15*	27.66 ± *0.57*	27.66 ± *1.15*	33.00 ± *1.00*	39.33 ± *0.57* **	*15.00 ± 0.00* **	*0.00*	*<0,001*
	***S***	***S***	***S***	***S***	--	***S***	***S***	--	*R*	
clinical strain 5	65.33 ± *0.57* **	31.00 ± *1.73*	27.33 ± *0.57* **	23.33 ± *0.57* **	28.33 ± *0.57*	31.66 ± *0.57*	38.00 ± *1.73* **	*14.66 ± 0.57* **	*0.00*	*<0.001*
	***S***	***S***	***S***	***S***	--	***S***	***S***	--	*R*	
clinical strain 6	56.33 ± *1.52* **	31.00 ± *0.00*	27.66 ± *0.57*	22.66 ± *0.57* **	27.00 ± *1.00*	31.66 ± *0.57*	34.66 ± *0.57* **	*15.33 ± 0.57* **	*0.00*	*<0.001*
	***S***	***S***	***S***	***S***	--	***S***	***S***	--	*R*	
*ATCC 43717 (Suny aB75)*	69.66 ± *0.57* **	40.33 ± *0.57* **	25.66 ± *0.57*	28.33 ± *0.57*	29.33 ± *0.57*	30.66 ± *0.57*	33.66 ± *0.57* **	*24.66 ± 0.57*	*0.00*	*<0.001*
	***S***	***S***	***S***	***S***	--	***S***	***S***	--	*R*	
ATTC 43718 Y4	65.33 ± *0.57* **	25.33 ± *0.57*	34.33 ± *0.57*	28.66 ± *0.57*	28.66 ± *1.15* **	31.00 ± *1.00* **	33.33 ± *0.57*	*24.33 ± 0.57*	*0.00*	*<0.001*
	***S***	***S***	***S***	***S***	--	***S***	***S***	--	*R*	
HK1651 (JP2)	67.66 ± *1.52* **	28.33 ± *0.57*	27.66 ± *0.57*	28.33 ± *0.57*	27.33 ± *0.57*	34.33 ± *0.57*	32.00 ± *1.73*	*23.66 ± 0.57* **	*0.00*	*<0.001*
	***S***	***S***	***S***	***S***	--	***S***	***S***	--	*R*	
*HK 921* (JP2)	37.00 ± *1.73*	29.66 ± *0.57*	27.66 ± *0.57*	29.00 ± *0.00*	25.66 ± *0.57*	34.66 ± *0.57*	29.33 ± *1.15*	*20.33 ± 0.57* **	*0.00*	*<0.001*
	***S***	***S***	***S***	***S***	--	***S***	***S***	--	*R*	
HK1605 (non JP2)	46.00 ± *1.00* **	29.33 ± *0.57*	22.66 ± *0.57* **	26.66 ± *1.15* **	29.33 ± *1.15*	29.33 ± *1.15* **	32.00 ± *1.00* **	*19.66 ± 0.57* **	*0.00*	*<0.001*
	***S***	***S***	***S***	***S***	--	***R***	***S***	--	*R*	

Calculation of Inhibition Zone Diameter includes the diameter of the well (6mm). * Mean ± Standard deviation; R: resistant;S: susceptible; EO: essential oil. ** *p* < 0.01: the inhibition zone diameter of a group (EO or antimicrobials) vs. the inhibition diameters of all other groups for each strain. ^1^ Diameter of inhibition zones, including diameter of well 6 mm, ^2^ Amoxicillin + Clavulanic Ac, ^3^ Amoxicillin, ^4^ Doxycycline; ^5^ Spiramycine; ^6^ Ciprofloxacine; ^7^ Minocycline; ^8^ Vancomycin; ^9^ Metronidazol. ^10^ Breakpoint is missing for Amoxicillin; however, susceptibility can be interred from ampicillin, for which the breakpoint is: S ≥ 16 and R< 16 mm. ^11^ breakpoint is missing for Doxycycline; however, isolates susceptible to tetracycline are also susceptible to Doxycycline. Breakpoint for tetracycline is: S ≥ 25 and R< 22.

**Table 3:** Minimum inhibitory concentrations (MIC) and minimum bactericidal concentrations (MBC) of *O. vulgare* EO for the selected *A. actinomycetemcomitans* strains.

A. *Actinomycetemcomitans* Strains	*O. vulgare*EO		
	MIC(μg/mL) *	MBC (μg/mL) *	MIC/MBC *
clinical strain 1	1.00 ± *0.43*	1.51 ± *0.00*	0.99 ± *0.87*
clinical strain 2	0.49 ± *0.21*	0.62 ± *0.21*	1.34 ± *0.58*
clinical strain 3	0.15 ± *0.51*	0.18 ± *0.00*	1.00 ± *0.00*
clinical strain 4	0.12 ± *0.05*	0.15 ± *0.51*	0.49 ± *0.05*
clinical strain 5	0.12 ± *0.05*	0.15 ± *0.05*	1.00 ± *0.86*
clinical strain 6	0.49 ± *0.21*	0.75 ± *0.00*	0.66 ± *0.29*
ATTC 43717 (Suny aB75)	0.09 ± *0.00*	0.09 ± *0.00*	1.00 ± *0.00*
Y4 ATTC 43718	0.18 ± *0.00*	0.18 ± *0.00*	1.00 ± *0.00*
HK1651 (JP2)	0.09 ± *0.00*	0.09 ± *0.00*	1.00 ± *0.00*
HK 921( JP2)	1.51 ± *0.00*	2.01 ± *0.87*	0.83 ± *0.28*
HK1605 (non JP2)	0.62 ± *0.21*	0.75 ± *0.00*	0.83 ± *0.29*

* Mean± standard deviation.

**Table 4:** Leukotoxicity (200 ng/mL) in presence of *Psidium guajava* or *O. vulgare* EO alone or in combination after 2 h incubation in cultures of PMA-differentiated THP-cells. Result is expressed as percent viable cells in relation to control cells (100%) based on quantitative neutral red uptake analyses. Mean ± SD of triplicate analyses.

Concentration (µL/mL)	Oil (%)	Guava (%)	Association oil and Guava (%)	*p*
0	99.99± 0.93 *	100 ± 0.93	100 ± 0.93	<0.001
4	93.82 ± 3.13 *	93.55 ± 0.98	96.27 ± 0.98	<0.001
8	95.73 ± 0.31 *	91.92 ± 2.72	106.33 ± 1.65	<0.001
16	98.99 ± 0.41 *	106.60 ± 6.14	90.01 ± 8.17	<0.001
31	102.52 ± 1.22 *	108.51 ± 10.02	105.79 ± 20.35	<0.001
62	78.86 ± 3.86 *	85.39 ± 7.24	93.55 ± 2.52	<0.001
125	59.28 ± 1.72 *	98.72 ± 6.81	84.30 ± 2.61	<0.001
250	−4.07 ± 0.31 *	93.01 ± 4.31	98.99 ± 9.99	<0.001

* Significant difference (*p* < 0.001).
